# Hybrid Double Enzyme Biocatalyst for Effective Degradation of Organic Pollutants

**DOI:** 10.1021/acsenvironau.5c00069

**Published:** 2025-07-09

**Authors:** Ani Vardanyan, Adam Ewerth, Gulaim A. Seisenbaeva

**Affiliations:** Department of Molecular Sciences, 8095 Swedish University of Agricultural Sciences, P.O. Box 7015, Uppsala 75007, Sweden

**Keywords:** tandem enzyme systems, enzyme cascade reaction, water treatment, organic pollutants, natural silicate matrices

## Abstract

This study focuses on the development of environmentally friendly double enzyme catalysts for the degradation of organic pollutants in water, addressing key environmental concerns. The hybrid tandem system of xanthine oxidase (XO) with horseradish peroxidase (HRP) is designed for sustainable water treatment by using a natural and eco-friendly silicate substrate, perlite, as a support for the enzyme cascade reaction. The catalytic process was optimized for environmental applications. XO-generated hydrogen peroxide through the oxidation of hypoxanthine, which then activated HRP to break down a variety of harmful pollutants, including industrial dyes, pharmaceuticals, and phenolic compounds. The system demonstrated high pollutant removal efficiency, reaching up to 100% in some cases, while maintaining catalytic stability across a range of temperatures and pH values. Importantly, the biocatalytic system addressed secondary pollution—a common issue in conventional treatments. Thus, uric acid, a potential byproduct of the XO catalytic action, was degraded by HRP, preventing the accumulation of harmful byproducts in purified water. This research highlights the potential of the tandem XO-HRP enzyme cascade as a green, efficient, and sustainable solution for water purification, offering an environmentally responsible alternative to traditional methods that often contribute to further contamination.

## Graphical abstract

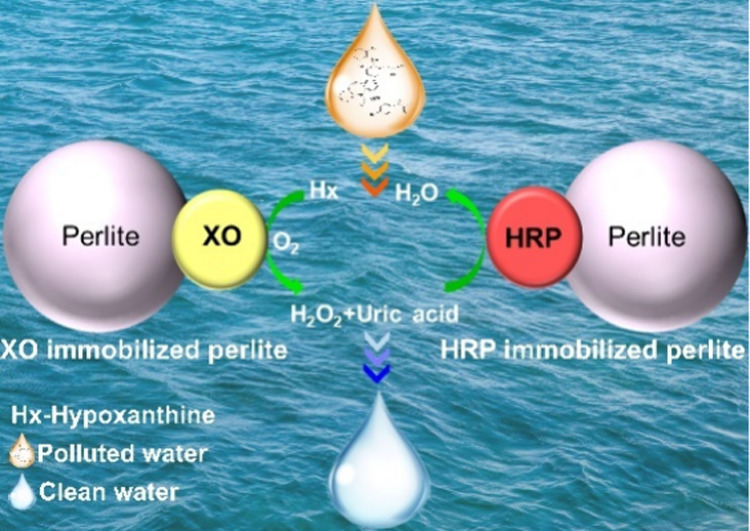

## 1 Introduction

The engineering of multienzyme systems has recently emerged as a rapidly expanding field of research, driven by the crucial role the enzymes play in facilitating complex metabolic processes in living cells.^1,2^ Many metabolic pathways operate via enzyme cascades, where the product of one enzyme serves as the substrate for the next, as seen in numerous biological systems, including those in humans, animals, and the natural environment.^2^ These cascades occur within the same cellular compartment, bringing enzyme active sites in close proximity to one another, which minimizes the diffusion of intermediates and enhances the overall efficiency and specificity of reactions.^3^ This spatial organization has inspired the development of artificial multienzyme systems, which have found broad applications in scientific and industrial processes, such as pharmaceutical synthesis, biofuel production, and fine chemicals manufacturing.^4^ Examples include one-pot reactions like carbohydrate synthesis,^5,6^ polymer production,^7^ and cellulose hydrolysis via synergistic action of endoglucanase, cellobiohydrolase, and β-glucosidase.^8^ Such multienzyme reactions offer numerous benefits in industrial applications, including fewer unit operations, shorter cycle times, smaller reactor volumes, improved space-time yields, and reduced waste generation.^1,9^

In recent years, there has been an increasing emphasis on the utilization of enzymes, not only for synthetic applications but also in the context of water treatment.^10–13^ Among various strategies developed for the removal of organic pollutants, the enzyme-catalyzed treatments are considered a favorable option owing to their notable biocatalytic activity, selectivity, and environmental friendliness. One of the most studied groups of enzymes capable of catalyzing the degradation of different organic molecules, such as phenolic compounds, dyes, and pharmaceuticals, includes peroxidases.^14^ However, these enzymes require hydrogen peroxide (H_2_O_2_) for initiation, making the process not entirely environmentally friendly. Furthermore, high concentrations of H_2_O_2_ may lead to irreversible inactivation of hem peroxidases, causing reduced reaction efficiency.^15^ The utilization of enzyme cascades offers a promising approach to address these challenges by facilitating the controlled generation of hydrogen peroxide through another enzyme.^16^ Nevertheless, limited studies have explored the application of enzyme cascades for water treatment purposes.

Previous studies have focused on combinations like horseradish peroxidase (HRP) and glucose oxidase (GOD), with promising results for the degradation of different organic pollutants.^17–19^ HRP can effectively catalyze the oxidation of a broad range of aqueous–organic substrates, especially phenolic compounds, in the presence of H_2_O_2_.^20^ GOD, on the other side, has shown great potential for the production of H_2_O_2_ and gluconic acid (GA) via the aerobic oxidation of β-d-glucose.^21^ Gao et al. synthesized magnetic combined cross-linked enzyme aggregates of GOD and HRP and tested the combination of these enzymes on the removal of direct black (DB38).^22^ The results demonstrated a higher removal rate (92%) than that of free enzymes (47%). In another study by Farhadi et al., GOD and HRP were coimmobilized into metal–organic frameworks (MOFs), which not only provided an optimum microenvironment for both enzymes but also provided a protective shield for the enzymes.^23^ The prepared biocatalyst was then tested for the removal of bisphenol A (BPA) and demonstrated 80% degradation of the organic compound at an initial concentration of 20 μg/mL. Another recent work by Li et al. demonstrated the use of the same enzymes (HRP and GOD) for the degradation of Alizarin Green (AG) and showed nearly 2.7 times higher reaction rate than that of a single-enzyme system (HRP/H_2_O_2_).^17^

Inspired by these promising results, we aimed to develop novel double enzyme systems that mimic biological catalytic pathways while enhancing environmental sustainability. We selected xanthine oxidase (XO), a key enzyme in purine catabolism, to be combined with HRP in a cascade system for the degradation of organic pollutants in water. Previously, Wei et al. utilized a combination of XO and HRP enzymes to develop an indicator for the one-step detection of fish freshness.^24^ In their study, XO and HRP were immobilized on nitrocellulose membranes with 3,3′,5,5′-tetramethylbenzidine to produce a colorimetric signal.^24^

To the best of our knowledge, this is the first attempt to employ an XO-HRP enzyme cascade in situ for the degradation of organic pollutants in water. We selected this enzyme pairing based on the complementary catalytic roles of the enzymes: XO generates hydrogen peroxide through the oxidation of hypoxanthine, while HRP utilizes the generated H_2_O_2_ necessary for its activation and subsequent oxidation of pollutants. Additionally, based on previous literature indicating that HRP can oxidize uric acid, which is a byproduct of XO activity, we hypothesized that HRP could further remove uric acid, potentially mitigating secondary pollution. In this way, the two enzymes operate not only in a traditional cascade but also in a more complete reaction cycle, where all intermediates and byproducts are further processed. We immobilized both enzymes on perlite, which is a cost-effective and environmentally friendly natural silicate. Building on our previous work in core–shell enzyme immobilization on natural silicates, we aimed to create a reusable and robust enzymatic system for the efficient degradation of dyes and other organic compounds in water.^13^

## 2 Materials and Methods

### 2.1 Materials

For the synthetic procedures, the following reagents have been used: tetraethoxysilane (TEOS), Sigma-Aldrich Sweden AB, CAS no. 78–10–4, catalog number 8.00658, purity: synthesis grade, ammonium fluoride, Sigma-Aldrich Sweden AB, CAS no. 12125–01–8, catalog number 338869, purity: ≥99.99%, hydrogen peroxide, Sigma-Aldrich Sweden AB, CAS no. 7722–84–1, catalog number 1.08600, 2,2′-azino-bis­(3-ethylbenzothiazoline-6-sulfonic acid) diammonium salt (ABTS), Sigma-Aldrich Sweden AB, CAS no. 30931–67–0, catalog number A1888, purity: ≥98%, perlite, Impecta Fröhandel, Sweden.

The enzymes were purchased from Sigma-Aldrich with their enzyme activity details specified on the bottles: HRP, CAS 9003–99–0, catalog no. 77332, 156 U/mg; xanthine oxidase, CAS no. 9002–17–9, 1.02 U/mg, catalog number X4376. Following organic compounds for water treatment assays were used: Diclofenac sodium salt (DFC), Sigma-Aldrich Sweden AB, CAS no. 15307–79–6, purity: ≥98% (TLC); phenol, Sigma-Aldrich Sweden AB, CAS no. 108–95–2, purity: analytical standard ACS; acetaminophen (paracetamol, PC), Sigma-Aldrich Sweden AB, CAS no. 103–90–2, purity: analytical standard ACS; rhodamine B (RhB), Sigma-Aldrich India, CAS no. 81–88–9, catalog number R6626, purity: ≥95%; bromophenol blue (BpB), Thermo Scientific Chemicals UK, CAS. no. 115–39–9, catalog number A18469, purity: ≥95%.

### 2.2 Methods

#### 2.2.1 Preparation of Core–Shell Enzyme Biocatalysts

The core–shell perlite particles were prepared according to the previous literature.^13,25^ Briefly, 150 mg of perlite was suspended in 10 mL of enzyme solution in water with specific enzyme concentrations (20 U/mL for HRP and 0.5 U/mL for XO), allowing the enzyme to adsorb overnight. Afterward, 25 mL of ethanol, 15 mL of water, and 0.2 mL of 1% NH_4_F in water were added. To get the silica shell, 4 mL of TEOS in 5 mL of ethanol (EtOH) was added dropwise over 30 min. After several hours, the solution became viscous and transformed into a gel. The mature gel was sequentially washed three times with water and three times with ethanol (each wash involving 30 min of gentle shaking), and the supernatants were collected after each step. Enzyme concentration in these washing solutions was measured by activity assays to evaluate enzyme leaching and calculate the immobilization efficiency.

#### 2.2.2 Characterization

Particles were morphologically characterized by scanning electron microscopy using a Hitachi (Tokyo, Japan) Flex-SEM 1000-II environmental scanning electron microscope at an acceleration voltage of 5 kV, a spot size of 20, and a working distance of 5 mm. Elemental analyses of surfaces were performed using energy-dispersion spectroscopy (EDS), applying the electron microscope mentioned above, combined with an AZtecOneXplore EDS detector by Oxford Instruments (UK). For each sample in EDS analyses, at least five different areas were studied, and an acceleration voltage of 20 kV, a spot size of 50, and a working distance of 10 mm were used. The average value was then calculated and given as the relative content of the elements.

UV–vis measurements were performed using a Multiskan Sky High (Thermo Fisher Scientific, Waltham, MA, USA) apparatus and standard 96-well plates (for HRP activity assay) or quartz cuvettes (for the XO activity assay).

Concentrations of organic pollutants and degradation kinetics were obtained by using NMR and UV–vis spectroscopy. For UV–vis measurements, the absorption was recorded between 200 and 800 nm, and the maximum absorption wavelength was determined accordingly. The NMR experiments were acquired on Bruker Avance III 600 MHz spectrometers, operating at 14.1 T, that were equipped with a cryo-enhanced QCI-P probe at a temperature of 298 K. Chemical shifts were referenced to D_2_O at 0.0 ppm. The data were processed and analyzed with the TopSpin 4.3.0 (Bruker) program. For all experiments, after removal of the biocatalysts, the sample solution was filtered through 0.2 μm cellulose membranes. The final water solution, 500 μL, contained 10% of D_2_O.

Fourier-transform infrared (FTIR) spectra of biocatalysts were recorded as KBr pellets using a demountable cell with KBr glasses on a PerkinElmer Spectrum 100 instrument.

Thermogravimetric analyses (TGA) were carried out using a PerkinElmer (Waltham, MA, USA) Pyris 1 instrument in an air atmosphere at a heating rate of 5 degrees/min in the 25–900 °C interval.

Powder X-ray diffraction (PXRD) data were obtained on a Bruker D8 QUEST ECO diffractometer equipped with a Proton III area detector and graphite-monochromated Mo Kα (λ = 0.71073 Å) radiation source. Data was processed with the EVA-12 software package.

#### 2.2.3 Enzyme Activity Assays

The activity of free HRP was performed by monitoring the oxidation of ABTS according to the colorimetric procedure reported previously in the literature.^26^ An ABTS stock solution (0.2 mM) was prepared in potassium phosphate buffer (pH 6.5 and 0.1 M). The assay was performed in a 96-well plate, where each well contained 90 μL of the ABTS stock solution and 10 μL of the enzyme solution (0.2 U/mL). Prior to use, 30 μL of 3.6% (≈1.1 M) hydrogen peroxide was added to 1 mL of the ABTS stock solution to activate the enzyme. In the final assay mixture, the concentrations of ABTS and hydrogen peroxide were 0.18 and 31.5 mM, respectively. The reaction was conducted at room temperature (23 °C). One unit of enzyme activity was defined as the amount of enzyme required to oxidize 1 μmol of ABTS (molar extinction coefficient of ABTS•^+^ ε_410_ = 36,000 M^–1^ cm^–1^)^27^ per minute per unit volume and is expressed in U/mL.

For free XO activity measurement, the method developed by Jorgensen was utilized using hypoxanthine to initiate the oxidation reaction.^28^ The rate of hypoxanthine transformation to uric acid was monitored by UV–vis spectroscopy at 293 nm (molar extinction coefficient of uric acid ε_293_ = 12,300 M^–1^cm^–1^)^28^ using a quartz cuvette with a 1 cm path length. For this purpose, 5 mg hypoxanthine was dissolved in 10 mL Milli-Q water by heating the solution to 50 °C, resulting in a stock solution of 0.5 mg/mL (≈3.7 mM). This solution was subsequently diluted 10-fold with potassium phosphate buffer (pH=6.5, 0.1 M), yielding a final hypoxanthine concentration of approximately 0.37 mM in the assay mixture. The assay was performed at room temperature (23 °C) in an open 3 mL quartz cuvette containing 2.9 mL hypoxanthine solution and 100 μL enzyme solution (0.2 U/mL). All reactions were conducted under ambient conditions with sufficient air exposure to ensure the oxygen availability for the reaction. One unit of enzyme activity was defined as the amount of enzyme required to oxidize 1 μmol of hypoxanthine per minute per unit volume and is expressed in U/mL.

All enzyme assay measurements were performed in triplicate, and the standard deviation was less than 5% of the mean.

#### 2.2.4 Enzyme Cascade Stability

The stability of both free and immobilized HRP-XO tandem enzyme cascades was evaluated by monitoring the degradation of RhB under varying pH and temperature conditions. Previous studies have demonstrated that HRP can effectively degrade RhB through oxidative pathways involving H_2_O_2_, supporting its use as a reliable model compound for activity assessments.^29,30^ To activate the cascade, 1 mL of hypoxanthine solution (0.05 mg/mL, ≈0.37 mM) was added to each test system, which ensured adequate in situ hydrogen peroxide generation by XO, with the hypoxanthine amount selected to provide sufficient stoichiometric support for HRP-catalyzed pollutant degradation. For immobilized systems, 50 mg each of HRP- and XO-loaded perlite–silica composite powders were suspended in 5 mL of RhB solution (100 μg/mL (≈0.21 mM), prepared in Milli-Q water) in 15 mL Falcon tubes. For free enzyme experiments, equivalent activity units corresponding to the immobilized enzymes were calculated, and 100 μL of HRP and XO enzyme solutions were added to the same volume and concentration of RhB solution to maintain consistent conditions across all tests.

For temperature stability experiments, the samples were incubated at 23, 30, 40, 50, and 60 °C using a digitally controlled hot plate. To evaluate pH stability, the pH of the dye solution was adjusted to 3, 4, 5, 6, 7, or 8 by using HNO_3_ and ammonia solutions. All reactions were carried out in Falcon tubes under ambient atmospheric conditions to ensure sufficient oxygen availability for XO catalysis. Samples were agitated on an orbital shaker for 24 h. After incubation, the supernatants were collected by centrifugation and analyzed by UV–vis spectroscopy to quantify the remaining RhB concentration.

#### 2.2.5 Comparative Pollutant Removal by Single Enzymes vs HRP-XO Tandem Enzyme System

The degradation efficiency of the double HRP-XO system was evaluated spectrophotometrically using two model dyes, BpB and RhB, by comparing the performance of individual free enzymes (HRP and XO), the free tandem enzyme cascade, and the immobilized tandem HRP-XO cascade. For each test, the enzyme amounts were normalized by activity units to ensure a consistent comparison across the systems.

Stock solutions of RhB and BpB were prepared in Milli-Q water at concentrations of approximately 100 μg/mL, corresponding to 0.21 mM for RhB and 0.15 mM for BpB. These stock solutions were subsequently diluted to obtain a range of working concentrations for catalytic performance evaluation. Specifically, RhB was tested at 1, 2.3, 4.5, 9.1, 45, and 91 μg/mL, while BpB was tested at 2, 5, 10, 50, and 100 μg/mL. For quantification, calibration curves were generated in the linear absorbance range using standard solutions of 0.5, 1, 2.5, 5, and 10 μg/mL for RhB, and 1, 2, 5, 10, and 20 μg/mL for BpB (Figure S2).

In all cascade experiments, hypoxanthine was added at the start to enable the in situ generation of H_2_O_2_ by XO. Specifically, 1 mL of a 0.05 mg/mL hypoxanthine solution (≈0.37 mM) was added to each reaction mixture. For the free HRP experiments, 150 μL of hydrogen peroxide (≈1.1 mM) was added at the start of the reaction to activate the enzyme.

For the immobilized systems, 50 mg of perlite-HRP or perlite-XO was used for single-enzyme experiments, and 50 mg of each (perlite-HRP and perlite-XO) was used in cascade reactions. For the free enzyme cascade, equivalent enzyme units were calculated and added together with hypoxanthine.

All reactions were carried out at room temperature (23 °C) in 15 mL Falcon tubes containing 5 mL of dye solution, under gentle agitation for 24 h. The tubes were not sealed to ensure sufficient oxygen availability for XO-catalyzed oxidation. After incubation, the samples were centrifuged to remove the solid catalyst, and the supernatants were analyzed by UV–vis spectroscopy. Absorbance maxima for RhB and BpB were recorded at 554 nm and 590 nm, respectively. The extent of dye degradation was calculated based on absorbance reduction and calibration curves.

To further demonstrate the applicability of the system to environmentally relevant targets, the immobilized HRP-XO cascade was tested for the degradation of common persistent organic micropollutants, including DFC (20 μg/mL ≈ 64 μM), PC (20 μg/mL ≈ 132 μM), and phenol (20 μg/mL ≈ 213 μM), which are frequently detected in surface and wastewater. Each 5 mL pollutant solution was treated with 50 mg of perlite-HRP and 50 mg of perlite-XO, along with 1 mL of hypoxanthine solution (0.05 mg/mL, ≈0.37 mM) to enable in situ H_2_O_2_ generation by XO. All experiments were conducted under the same conditions as previous assays (23 °C, 24 hours, pH = 5, gentle agitation). Since the UV–vis spectra of these compounds overlap with those of reaction components such as H_2_O_2_ and hypoxanthine, their degradation was instead assessed by ^1^H NMR spectroscopy, which allows more selective identification of structural changes.

Control experiments were also performed to evaluate the potential adsorption of dyes and micropollutants onto the support materials. In these experiments, 100 mg of perlite coated with a silica shell (prepared without enzyme incorporation) was incubated with 5 mL of RhB, BpB, DFC, PC, or phenol solution under identical conditions (23 °C, 24 hours, gentle shaking). These controls were used to distinguish between pollutant removal due to adsorption and enzymatic degradation. After incubation, all reaction mixtures were centrifuged for 10 min at 7000 × *g* to separate the solid biocatalyst. For pharmaceutical and phenol samples, the supernatants were further filtered through 0.2 μm cellulose membranes prior to ^1^H NMR analysis. For dye samples, UV–vis spectroscopy was performed directly on the centrifuged supernatants without filtration to avoid potential dye adsorption onto the filter membrane.

## 3 Results and Discussion

### 3.1 Fabrication and Characterization of Core–Shell-Immobilized Biocatalysts

In our previous work, we reported a new core–shell immobilization technique using natural silicates as substrates for the immobilization of oxidase and peroxidase enzymes.^13^ Based on the previous study, perlite was selected for the fabrication of a double enzyme biocatalyst within a core–shell structure covered by a silica layer.

The immobilization process began by adsorbing each enzyme (HRP or XO) onto perlite in separate flasks, followed by silica shell formation by using Sol–Gel chemistry. The specific HRP concentration was chosen based on our previous findings, which identified optimized conditions that resulted in maximum encapsulation yield. Given that XO produces hydrogen peroxide, which is subsequently utilized by HRP, a lower concentration of XO (0.2 U/mL) was chosen relative to HRP (20 U/mL) to prevent excessive H_2_O_2_ generation that could potentially deactivate the peroxidase enzyme. Initial tests with free XO enzyme at concentrations of 0.2–0.5 U/mL exhibited high activity for the tandem HRP-XO cascade, leading us to select 0.2 U/mL of XO for subsequent immobilization experiments. The resulting biocatalyst powders were mixed in a 1:1 weight ratio after drying and stored at 4 °C for further use.

To confirm the successful immobilization of enzymes onto perlite, enzyme activity assays were first performed before and after the immobilization process (Figure S3). In addition, enzyme activity was measured in the washing solutions collected during the postimmobilization washing steps to account for any enzyme loss. Based on the measured activity in these supernatants, the amount of enzyme leached during washing was estimated to be approximately 1.1% for XO and 1.4% for HRP. These values were used to refine the final calculation of immobilized enzyme amounts, which were estimated to be 10.2 U/g (perlite) for XO and 1260 U/g for HRP. These activity-based measurements were conducted in triplicate and used as the primary method for quantifying enzyme loading.

As a complementary approach, thermogravimetric analysis (TGA) was used to qualitatively confirm the presence of organic content, such as protein, on the perlite-enzyme biocatalysts (Figure S4). The TGA curves showed that when the temperature increased from room temperature to 100 °C, a small mass loss occurred (6.8% for perlite-HRP and 9.4% for perlite-XO), which was attributed to the evaporation of residual water. Further heating from 100 to 500 °C resulted in additional weight loss (4.571% for perlite-HRP and 11.17% for perlite-XO), corresponding to the pyrolytic decomposition of organic material, including enzyme. Above 500 °C, continued mass loss was assigned to the decarbonization of remaining organic residues.

A reference TGA measurement using enzyme-free perlite subjected to identical treatment was included to distinguish the background thermal behavior. The control sample exhibited negligible mass loss under similar heating conditions (Figure S4c), confirming that the weight losses observed in enzyme-loaded samples result from immobilized organic matter.

The occurrence of the silica shell was verified by FTIR analysis, which was applied to bare perlite and core–shell-immobilized samples (Figure 1). In all three samples characteristic peaks belonging to SiO_2_ were observed around 480, 800, and 1085 cm^–1^, corresponding to the δ­(Si–O–Si), υ­(Si–O–Si), and υ_as_(Si–O–Si) vibrations.^13,31^ Additionally, a new absorption band at 960 cm^–1^ emerged in both perlite-HRP and perlite-XO samples, corresponding to the stretching vibrations of the surface silanol (Si–OH) groups. This band confirms the formation of uncondensed silanols during the sol–gel process, consistent with previous reports on silica encapsulation under mild conditions.^13^ These silanol groups play a critical role in enzyme stabilization by forming hydrogen bonds with polar amino acid residues (e.g., Ser, His, and Asp) on protein surfaces.^32^ Such interactions create a hydrated microenvironment that mimics natural enzyme habitats, preserving tertiary structure and catalytic activity while mitigating denaturation under operational stresses.^33^

**Figure 1:**
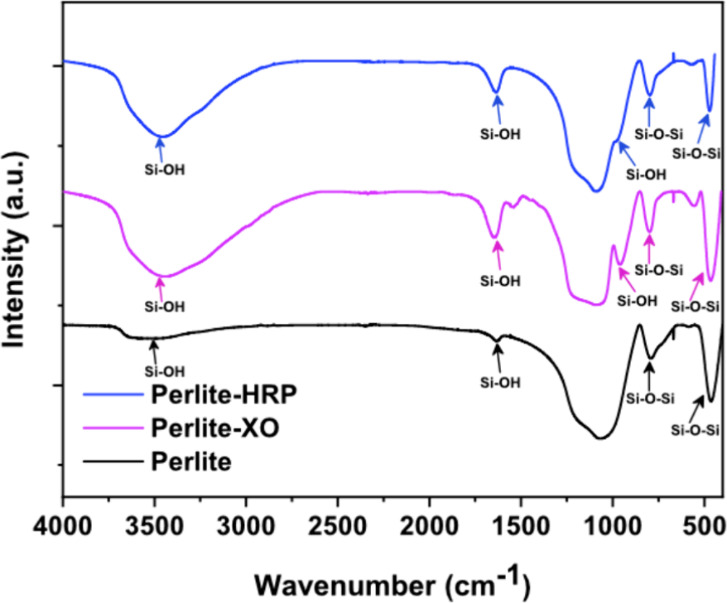
FTIR spectra of perlite before and after core–shell immobilization with HRP (blue) and XO (magenta) enzymes.

EDS measurements further confirmed the successful formation of a silica layer, with a notable increase in silicon content from 19 to 28% (atomic weight) on the surface of the enzyme-functionalized perlite (Figure S5). Simultaneously, the relative concentrations of elements characteristic of raw perlite, such as Fe, Na, K, and Ca, were reduced, indicating that the silica coating effectively masked the original mineral surface.

X-ray diffraction (XRD) analysis was conducted to examine the structural characteristics of perlite before and after core–shell immobilization. The resulting diffractograms (Figure S6) depict the amorphous nature of all three samples, indicated by the appearance of a broad peak positioned between 6 and 16° (2θ). In the native perlite sample, additional diffraction peaks were observed at approximately 11 and 13.5° (2θ), which were absent in the enzyme-adsorbed and core–shell-immobilized samples. These peaks were matched to albite, a sodium-rich feldspar, indicating the presence of minor crystalline phases in the raw material, which later disappear following the immobilization process, due to hydrolysis in water.^34,35^

### 3.2 Catalytic Performance of the Immobilized Tandem Enzyme Cascade

The catalytic activity of the immobilized tandem enzyme cascade was initially assessed by using the traditional ABTS oxidation assay. In this experiment, 100 mg of each immobilized enzyme was mixed in a 15 mL Falcon tube, and the reaction was initiated by adding 1 mL of hypoxanthine. During the reaction, xanthine oxidase (XO) converts hypoxanthine to hydrogen peroxide and uric acid. The hydrogen peroxide subsequently activates horseradish peroxidase (HRP), which oxidizes ABTS to ABTS^+^• (Figure 2B). This oxidation step can be monitored by UV–vis spectroscopy at 410 nm, as ABTS changes color from transparent to green, corresponding to the formation of ABTS^+^•.^26^ However, no color change was observed even after an extended incubation period. To verify whether uric acid was responsible for reducing the oxidized ABTS^+^•, we conducted a control experiment where uric acid was added to an already oxidized ABTS solution (HRP + H_2_O_2_) (see Supporting Information 2. Reduction of ABTS^+^• by uric acid). The results demonstrated a rapid decrease in absorbance at 410 nm upon uric acid addition, confirming its strong reducing effect. A control experiment in which water was added instead of uric acid showed no change in absorbance. These findings validate that uric acid, a byproduct of XO, interferes with the ABTS assay by reversing the ABTS^+^• oxidation (Figure S7).^36^ This phenomenon was further investigated by repeating the experiment with free enzymes. In this setup, equal volumes (100 μL) of each enzyme (20 U/mL for HRP and 0.5 U/mL for XO) were mixed in a 3 mL cuvette with 1 mL of hypoxanthine solution (0.05 mg/mL ≈ 0.37 mM) and 1 mL of ABTS solution (0.2 mM). As shown in the Supporting Information, the absorbance at 410 nm showed a rapid decline, starting from approximately 0.09. This indicates that ABTS^+^• was likely formed but rapidly reduced by uric acid before a significant accumulation could be detected. This result supports the hypothesis that uric acid interferes with ABTS-based assays in this cascade system (Figure S8).

**Figure 2:**
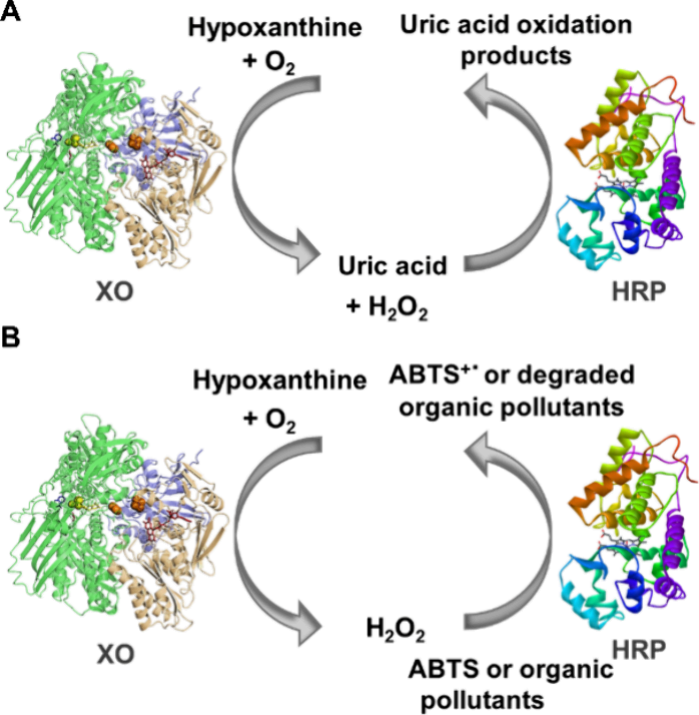
Schematic representation of substrate oxidation cascade by tandem XO-HRP system: oxidation of uric acid (A) and oxidation of ABTS or organic pollutants (B).

By reducing the volume of HRP enzyme solution 5-fold (from 100 μL to 20 μL, corresponding to 0.4 U instead of 2 U), it was possible to slow the rate of ABTS oxidation and monitor the reaction more clearly via UV–vis spectroscopy at 410 nm (Figure S9). All other components (XO, ABTS, and hypoxanthine) remained unchanged. The same approach was applied to the immobilized enzymes by reducing their quantities from 100 to 50 mg each (Figure S10). We observed that not only did the oxidation of ABTS slow down, but it also reached equilibrium without being affected by uric acid. We hypothesized that this may be due to the interaction between HRP and uric acid, where HRP could facilitate the oxidation of uric acid, thereby influencing the reaction dynamics (Figure 2 A). To test this theory, known concentrations of XO and hypoxanthine were mixed and incubated for 5 h to allow for uric acid production (see Supporting Information 1, Determination of Uric Acid in Water). Uric acid concentration was measured using UV–vis spectroscopy, after which HRP was added to the reaction mixture and incubated overnight. The results demonstrated that HRP completely degraded uric acid (Table S1). Similar findings have been previously reported by Padiglia et al.^37^ These observations confirmed that the cascade reaction relied on XO-generated H_2_O_2_ to initiate the HRP catalysis. Furthermore, the degradation of uric acid by HRP illustrated the complementary nature of the two enzymes, enhancing the completeness of the catalytic cycle.

While this adjustment enabled the calculation of cascade activity, it became evident that the ABTS assay was unsuitable for further activity and stability experiments due to significant interference from uric acid. Consequently, RhB was selected as an alternative model dye for subsequent stability experiments, offering a more reliable assessment of the double enzyme cascade’s catalytic performance.

### 3.3 Enzyme Cascade Stability

It is well-known that the pH and temperature of the catalytic environment are important factors influencing enzymatic reactions.^38–40^ Therefore, the enzyme activities of free HRP-XO and immobilized HRP-XO cascade systems were evaluated at different pH and temperature to obtain the optimal catalytic conditions. As shown in Figure 3A, the maximum enzyme activity of the free HRP-XO cascade was measured at room temperature (61% removal), and only 10% of the enzyme activity was retained at 60 °C. However, the optimal temperature of the immobilized HRP-XO cascade was extended to around 50 °C, and the enzyme activity of the HRP-XO cascade still remained about 65% even at 60 °C.

**Figure 3:**
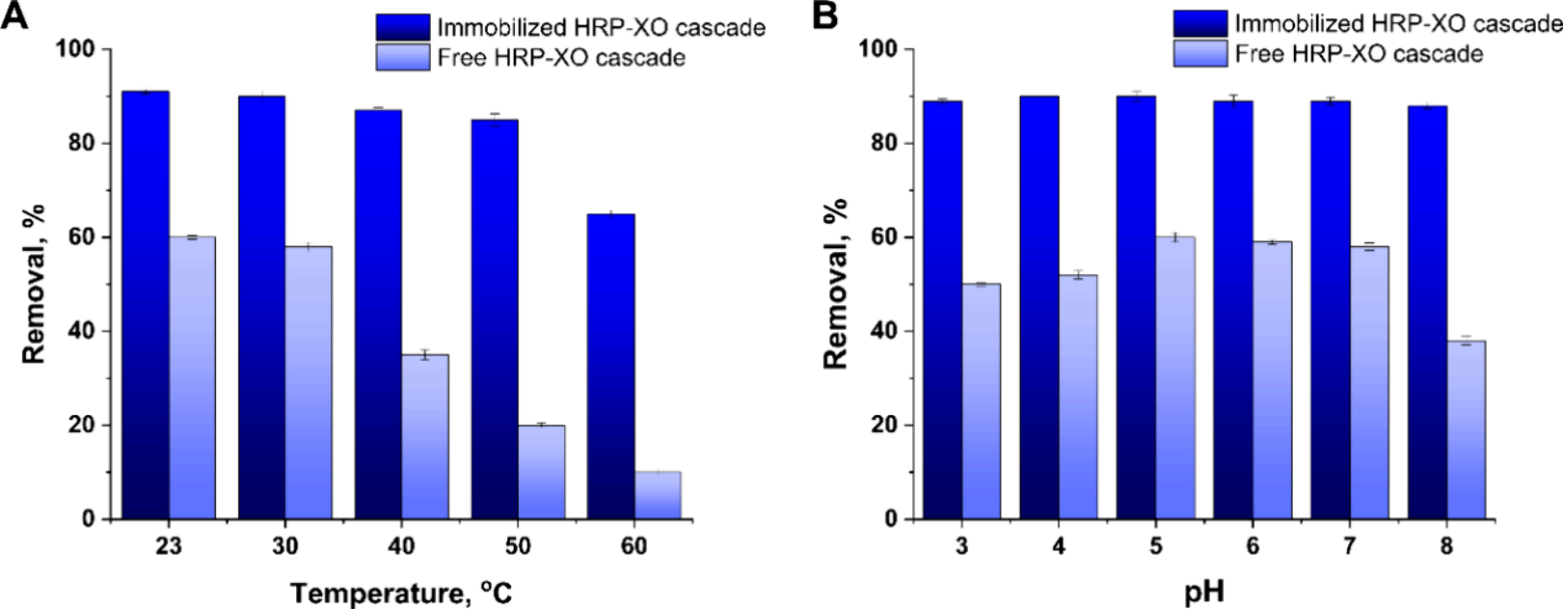
Stability of free and immobilized tandem HRP-XO cascades tested by RhB degradation at different (A) temperatures (23 to 60 °C) and (B) pH values (3 to 8). In all experiments, equal enzyme activity units were used for free and immobilized systems (10U for XO and 63U for HRP). RhB concentration was 100 μg/mL. Each data point represents the mean value of triplicate measurements with standard deviation highlighted as error bars.

The same phenomenon has also been observed in experiments with pH-dependent activity. These experiments for the free HRP-XO cascade and the immobilized HRP-XO cascade were conducted in the pH range from 3 to 8 (Figure 3B). The results suggested that the optimum reaction pH of the free tandem HRP-XO cascade was 5–6, whereas that of the immobilized tandem HRP-XO cascade could reach even to pH = 8.

The broader pH and temperature stability observed in the immobilized tandem HRP-XO cascade system can be explained by the protective silica matrix, which likely creates a stabilizing microenvironment around the enzymes. Such environments buffer against pH-induced protonation or deprotonation of catalytically essential residues and reduce the likelihood of protein unfolding by limiting solvent penetration and surface denaturation. In addition to pH stability, the thermal stability of immobilized enzymes is enhanced by the low thermal conductivity of the silica matrix,^41^ which reduces heat transfer to the enzyme and helps maintain structural integrity under thermal stress. Furthermore, the physical confinement provided by the porous silica shell restricts conformational flexibility and may prevent thermally induced unfolding by imposing steric constraints around labile regions of the protein. Such stabilization effects are well-documented for immobilized enzymes and are consistent with previous reports demonstrating enhanced resistance to harsh conditions through immobilization.^42^

### 3.4 Removal of Organic Pollutants by the Double Enzyme Cascade System

The efficacy of the double enzyme cascade was evaluated by quantifying the degradation rates of various organic pollutants, including RhB, BpB, PC, DFC, and phenol. These results were compared with those obtained from free enzymes and the free enzyme cascade system.

Figure 4 shows the removal efficiency of RhB (Figure 4A) and BpB (Figure 4B) at varying initial dye concentrations (1–100 μg/mL). HRP exhibited high activity toward BpB, achieving substantial degradation, even at elevated concentrations. When immobilized in a core–shell structure, the cascade’s activity decreased slightly, likely due to mass transfer limitations.^43^ The immobilized double enzyme cascade, however, showed higher activity compared to the free cascade reaction. It is important to note that in all cascade experiments, no external hydrogen peroxide was added. Therefore, the observed degradation could only occur if XO generated sufficient H_2_O_2_ to activate HRP. As XO alone did not catalyze pollutant degradation, this confirmed that the cascade was functionally active in situ.

**Figure 4:**
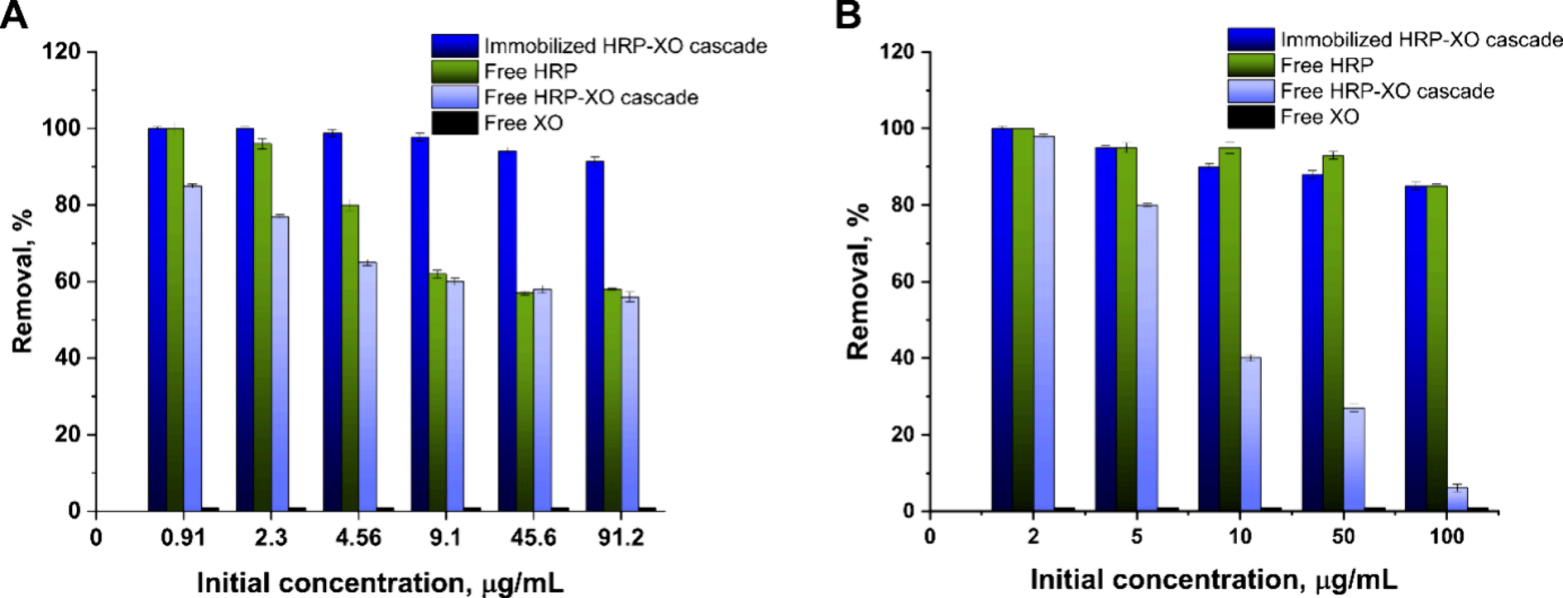
Removal of RhB (A) and BpB (B) by an immobilized and free tandem HRP-XO cascade compared to free HRP and XO activities. All experiments were carried out at room temperature (23 °C), pH = 6, and open to the air. Equal enzyme activity units were used for free and immobilized systems (10U for XO and 63U for HRP). Each data point represents the mean value of triplicate measurements with standard deviation highlighted as error bars.

The double enzyme cascade acted differently in the case of RhB. Similar to free HRP, the free double enzyme cascade showed a low oxidation rate at higher dye concentrations. However, the immobilized tandem cascade showed almost 100% removal of the dye, even at very high concentrations.

High degradation yield was achieved, as well, when testing the immobilized cascade on more resistant organic pollutants. We have tested three different compounds: DFC, PC, and phenol, which is one of the most common pollutants found in surface waters.^44–46^ The results showed about 95% degradation of PC (Figure 5A) and phenol (Figure S11) and 100% degradation in the case of DFC (Figure 5B).

**Figure 5:**
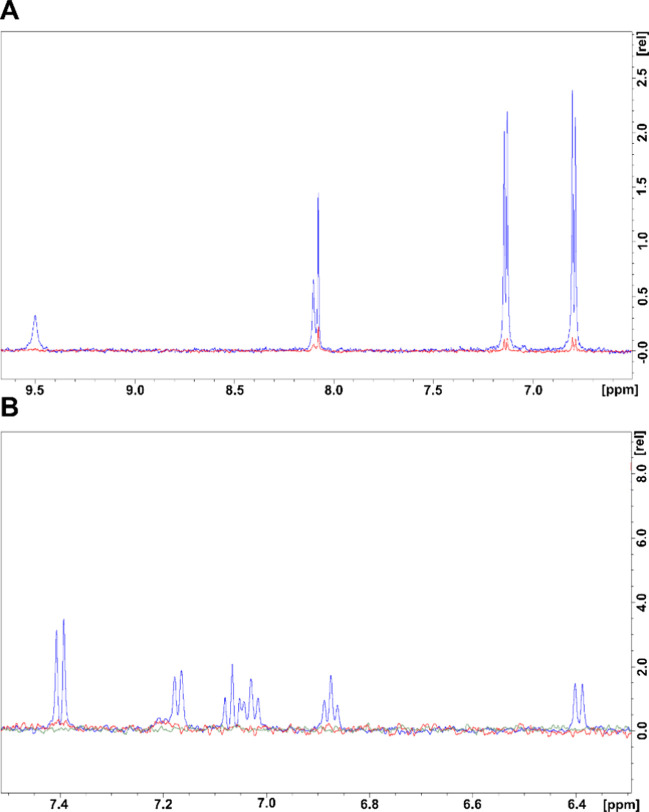
Degradation of PC and DFC by the core–shell-immobilized perlite-HRP-XO cascade: (A) initial PC (blue) and PC after 24 h of interaction with perlite-HRP-XO (red), (B) initial DFC (blue) and DFC after 24 h of interaction with perlite-HRP-XO (red). The NMR sample solution was H_2_O:D_2_O 90%:10%.

Control experiments with RhB and BpB were conducted using silica-coated perlite in the absence of enzymes to evaluate potential nonenzymatic adsorption. RhB showed approximately 15% removal, which was higher than the 5% observed for BpB (for the 100 μg/mL initial dye concentration). This increased RhB adsorption is likely due to electrostatic interactions between the cationic xanthene moiety of RhB and the negatively charged silica surface at pH 5.^47^ In contrast, BpB carries a net negative charge at pH 5,^48^ leading to reduced adsorption.

Similar control experiments were conducted for the persistent organic pollutants, where DFC, PC, and phenol were incubated with the silica-coated perlite in the absence of enzymes. Adsorption was minimal in all cases: PC showed negligible adsorption (<1%), phenol about 4%, and DFC around 3% (Figure S12). While weak hydrogen bonding or van der Waals interactions with surface silanol groups may occur, the observed pollutant removal in enzyme-treated systems can be attributed to enzymatic degradation rather than passive adsorption.

The degradation rate of different compounds depended on their structure and the enzyme specificity toward them.^49^ Kinetic experiments for dye degradation revealed a higher degradation rate for RhB compared to BpB, where more than 80% of RhB was removed in the first 5 min of the contact time with the immobilized tandem cascade (Figure 6). Degradation of BpB took a longer time, where about 70% of the dye was removed after 2 h, reaching its equilibrium in 3 h of contact time with the enzyme tandem cascade system.

**Figure 6:**
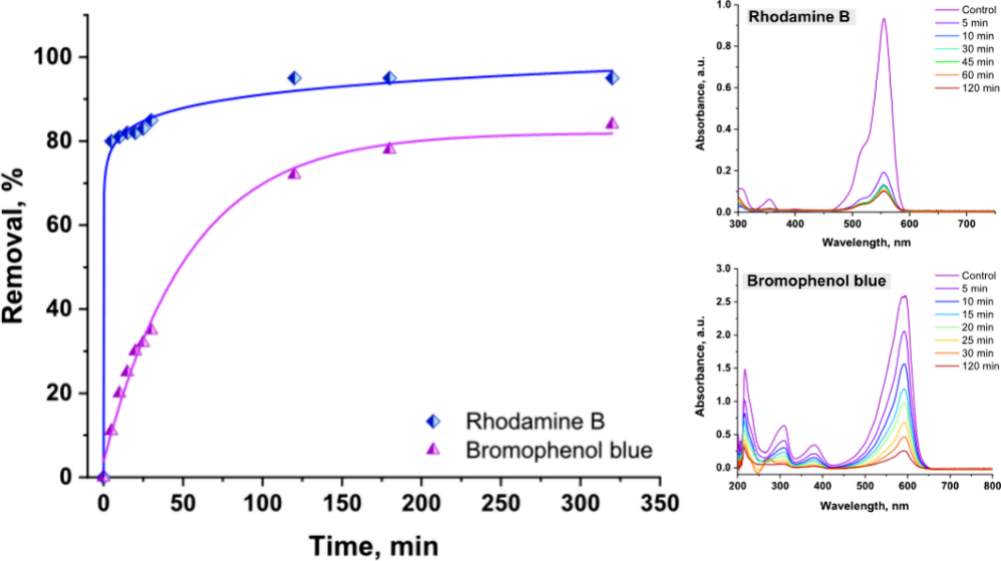
Effect of reaction time on the decolorization of RhB and BpB by an immobilized double enzyme cascade.

These results showed high activity for the immobilized double enzyme cascade, which could be ascribed to the enhanced stability of both enzymes.^11^ Studies have shown that immobilization can not only prevent enzyme leaching but also improve the enzyme activity by restricting their conformational flexibility and reducing the likelihood of denaturation under stress conditions. Covalent attachment^39^ or physical entrapment in matrices^25,50^ created multiple interaction points between the enzyme and the support, which minimized structural changes during catalysis and prevented enzyme aggregation or inactivation.^42^

These findings suggested that immobilization not only prevented enzyme leaching but also improved enzyme stability and activity, making this double enzyme cascade system a promising tool for sustainable water treatment technologies.

## 4 Conclusions

In this study, we developed and characterized a novel hybrid double enzyme system composed of XO and HRP, immobilized on a natural silicate substrate (perlite) within a silica core–shell structure. This biocatalyst efficiently degraded various organic pollutants in water, including industrial dyes (RhB and BpB), pharmaceuticals (PC and DFC), and phenolic compounds, achieving near-complete removal in several cases.

A key outcome of this work was the demonstration of a more complete reaction cycle, in which XO-generated hydrogen peroxide activated HRP for pollutant degradation, while HRP also removed the uric acid, a byproduct of XO. This set the system apart from traditional enzyme cascades that used to leave intermediate compounds untreated.

The enhanced thermal and pH stability of the immobilized enzymes further supported their suitability for practical environmental applications. These results indicated strong potential for using the tandem XO-HRP cascade as a robust green alternative to conventional water treatment technologies. Given the scalable and low-cost nature of perlite-based supports and the ability to operate under mild conditions, this approach offers a sustainable and practical alternative for wastewater remediation.

## Supplementary Material



## Notes

Procedure for determination of uric acid in water, protocol for reduction of ABTS^+^• by uric acid, calibration curve for uric acid, UV–vis absorbance results of uric acid concertation, calibration curves used for dye quantification, enzyme activity measurement by UV–vis before and after immobilization in perlite particles, TGA analysis of immobilized XO and HRP on core–shell perlite particles and pure perlite, EDS analysis of perlite before and after core–shell immobilization, X-ray diffraction (XRD) pattern of perlite before and after enzyme immobilization, activity of free enzyme cascade measured by ABTS test, activity of free enzyme cascade using lower HRP concentration measured by ABTS test, activity of immobilized enzyme cascade measured by ABTS test, degradation of phenol by core–shell-immobilized perlite-HRP-XO cascade; control experiments with silica-coated perlite (without enzymes) (PDF)
