# Supplementary material for: Hybrid Double Enzyme Biocatalyst for Effective Degradation of Organic Pollutants
Source: ACS Environ Au. 2025 Jul 9;5(5):501–10. doi: 10.1021/acsenvironau.5c00069 (PMC12447223; doi:10.1021/acsenvironau.5c00069)
Supplement: Supplementary file 1 [file vg5c00069_si_001.pdf]

Supporting information

Hybrid double Enzyme Biocatalyst for Effective Degradation of Organic Pollutants

Ani Vardanyan, Adam Ewerth, Gulaim A. Seisenbaeva*

a Department of Molecular Sciences, Swedish University of Agricultural Sciences, P.O. Box 7015, 75007 Uppsala, Sweden

1. **Determination of uric acid in water**

Uric acid quantification was performed using the Folin-Denis method, which employs phosphotungstic acid as a colorimetric reagent to measure uric acid in blood serum[1]. In this study, we modified this method to quantify uric acid produced by the enzyme (XO) in aqueous media. For this purpose, XO (0.2%U/mL) and hypoxanthine (0.05%mg/mL) were mixed in equal volumes (1%mL each) and incubated at room temperature for 5 hours to allow uric acid formation. To assess whether HRP could degrade the uric acid, 100%µL of HRP solution (0.2%U/mL) was then added to the mixture and incubated overnight at room temperature.

Following incubation, 1%mL of the reaction solution was combined with 1%mL of 1% phosphotungstic acid (prepared in Milli-Q water) and 1%mL of 0.5%M NaOH to maintain alkaline conditions. The blue color that developed over 15 minutes was measured at 710%nm using UV-Vis spectrophotometry. A standard calibration curve was generated using uric acid

[1]Folin, O.; Denis, W. On Phosphotungstic-Phosphomolybdic Compounds As Color Reagents. *Journal of Biological Chemistry* **1912**, *12* (2), 239–243.

solutions ranging from 0.01 to 0.1%mg/mL (Figure S1). This setup enabled quantification of uric acid before and after HRP treatment, confirming its enzymatic degradation.

[1]Folin, O.; Denis, W. On Phosphotungstic-Phosphomolybdic Compounds As Color Reagents. *Journal of Biological Chemistry* **1912**, *12* (2), 239–243.

2. Reduction of ABTS$^{+\cdot}$ by uric acid

To evaluate the reducing effect of uric acid on oxidized ABTS^{+}•, a reaction mixture was prepared containing 900%μL of ABTS solution (0.2%mM in 0.1%M potassium phosphate buffer, pH 6.5), 30%μL of hydrogen peroxide (3.6%), and 10%μL of horseradish peroxidase (0.2%U/mL). After allowing the green color to develop, indicating successful oxidation of ABTS, 100%μL of uric acid solution (0.5%mg/mL) was added to the mixture. The absorbance at 410%nm was monitored over a period of 5%minutes to track the reduction of ABTS^{+}•. A control experiment was conducted in parallel under identical conditions, where 100%μL of Milli-Q water was added instead of uric acid.

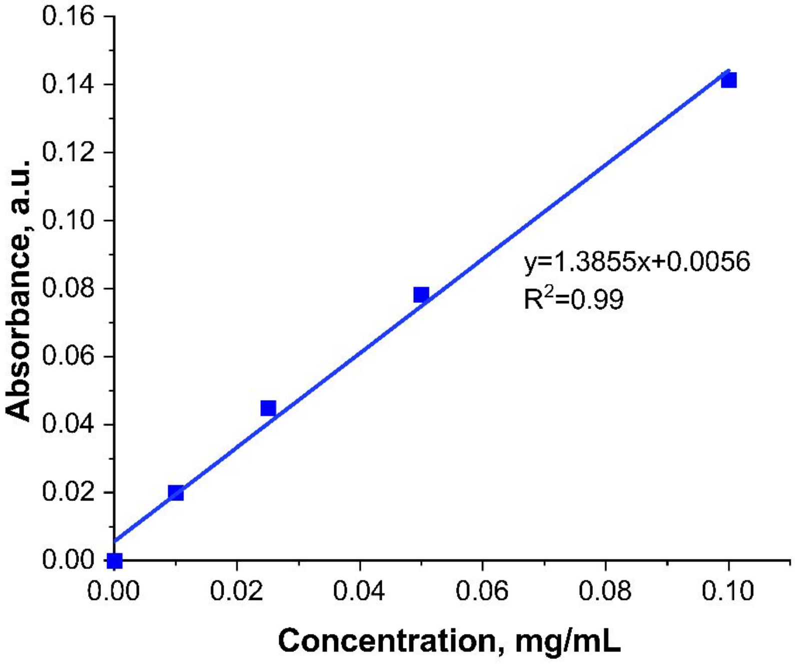

Figure S1. Calibration curve for uric acid. Absorbance was measured at 710 nm in a standard cuvette (path length: 1 cm).

Table S1. Uric acid concentration in XO-Hypoxanthine reaction before and after HRP addition

Sample	Absorbance	Calculated uric acid concentration (mg/mL)
**XO+Hypoxanthine**	0.0125	0.005
**XO+HRP+Hypoxanthine**	0.004	0 (-0.0012)

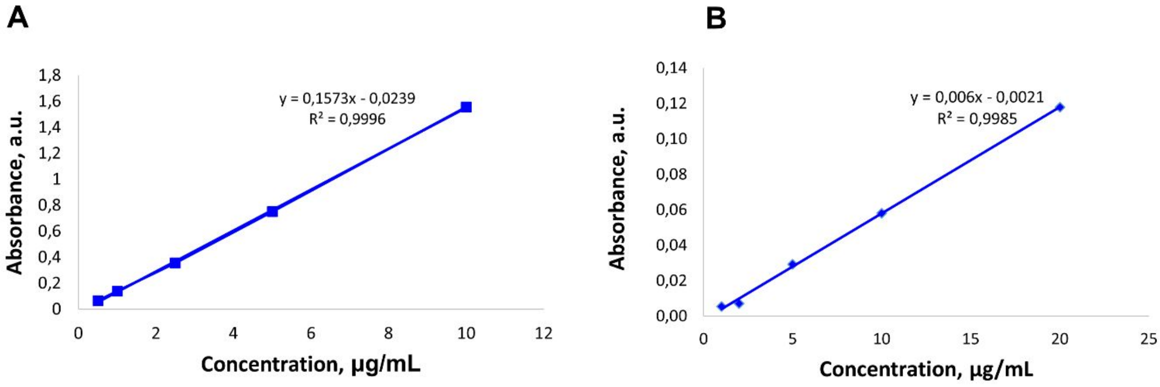

Figure S2. Calibration curves used for dye quantification: A. Rhodamine B (RhB) B. Bromophenol blue (BpB). Absorbance was recorded at 554%nm for RhB and 590%nm for BpB.

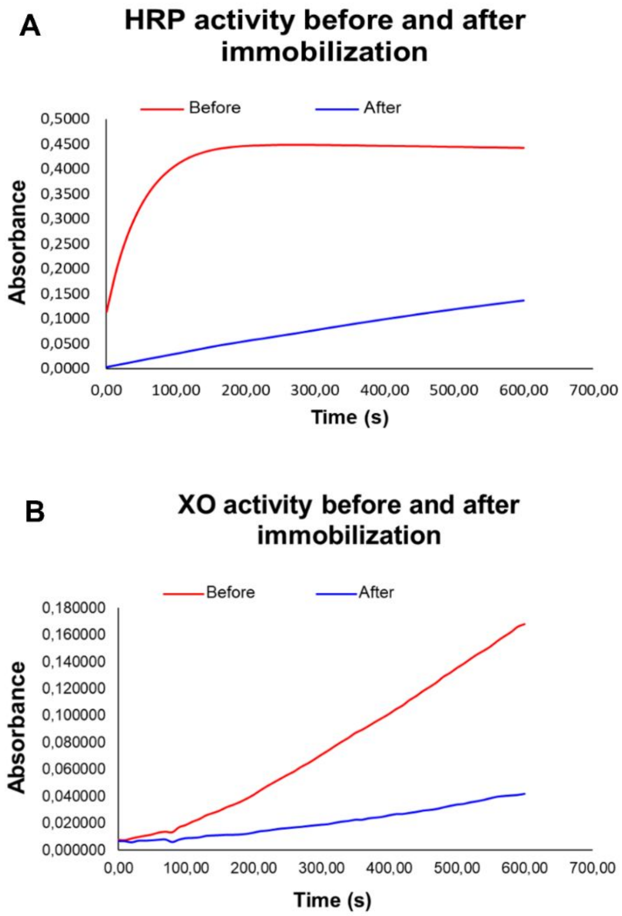

Figure S3. UV-Vis enzymatic activity measurements before and after immobilization in perlite particles: (A) HRP, measured through ABTS assay at 420 nm wavelength in a 96 well plate (path length: 0.3 cm). (B) XO, measured through formation of uric acid monitored at 293 nm wavelength, in a quartz cuvette (path length: 1 cm).

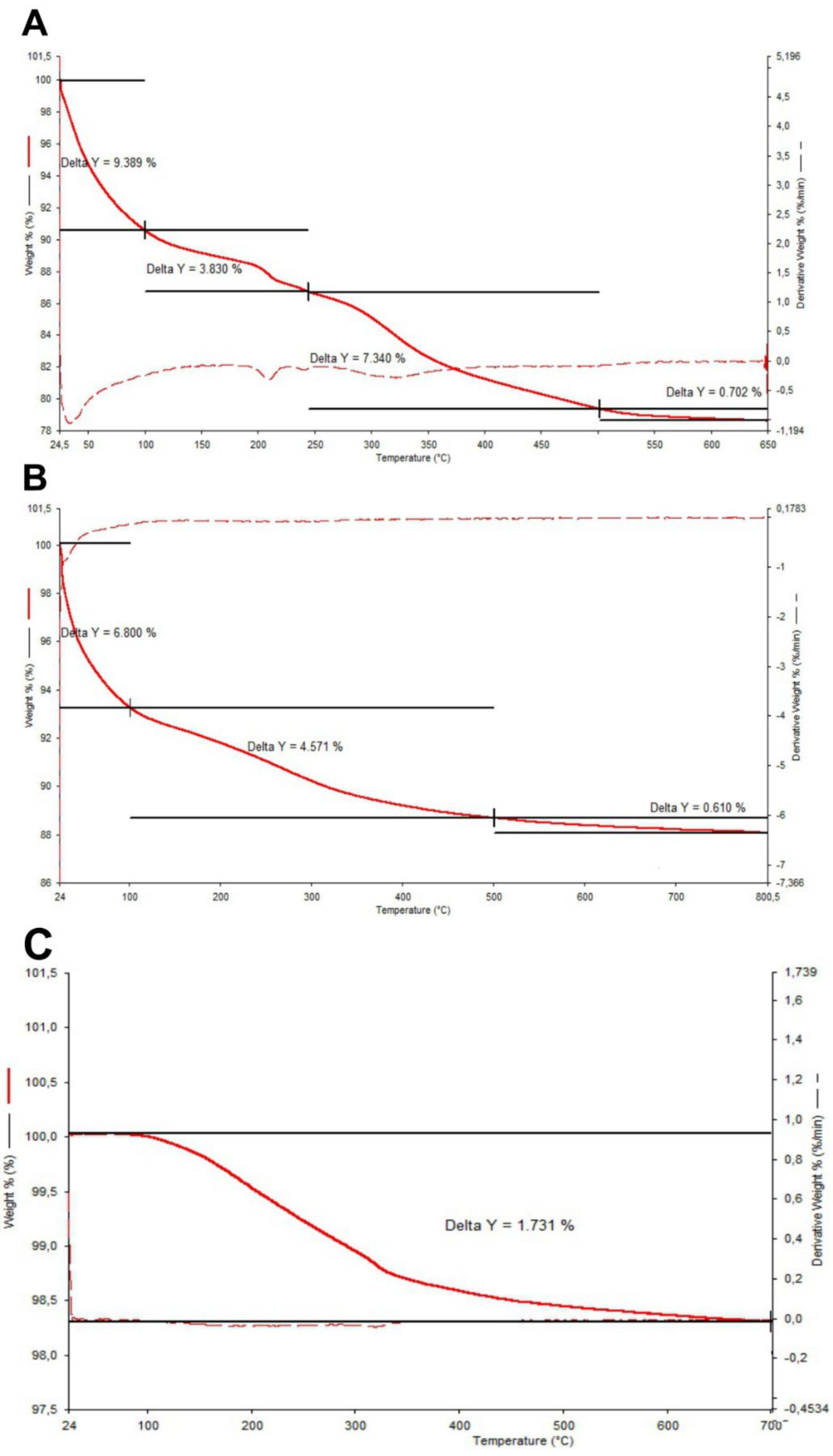

Figure S4. TGA analysis of immobilized XO (A) and HRP (B) on core shell perlite particles and perlite with silica shell without enzymes (C).

A

Map Sum Spectrum	Perlite	Perlite-SiO_2
Element	Atomic %	Atomic %
Fe	0.44	0.05
O	57.57	65.50
Si	19.08	28.10
Na	1.88	0.39
K	1.40	0.34
Ca	0.30	0.07
C	10.86	3.42
N	4.60	2.13
Total	100.00	100.00

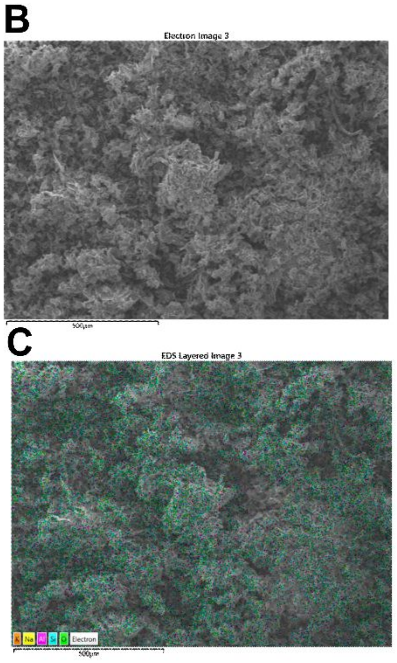

D

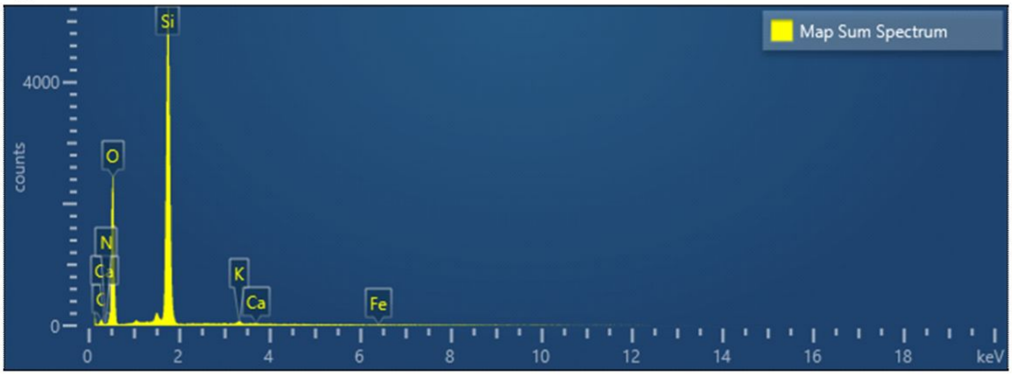

Figure S5. EDS analysis of perlite before and after core-shell immobilization: Silica content measured by EDS analysis before and after immobilization (A), Electron image of measured perlite site (B), EDS layered image of perlite after silica shell formation (C), EDS map spectrum (D).

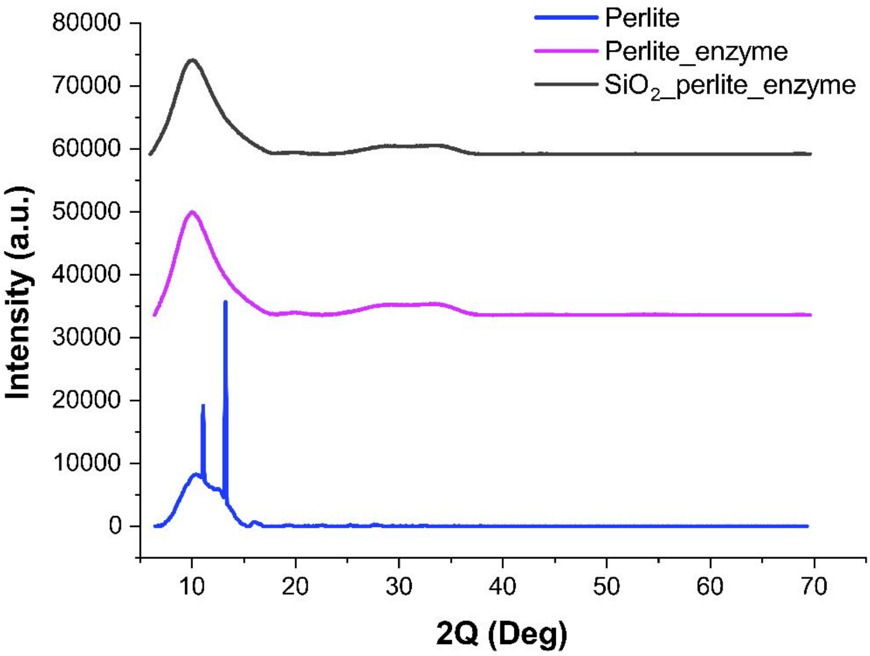

Figure S6. X-ray diffraction (XRD) patterns of perlite (blue), perlite after HRP enzyme immobilization (magenta), and core-shell immobilized perlite (black).

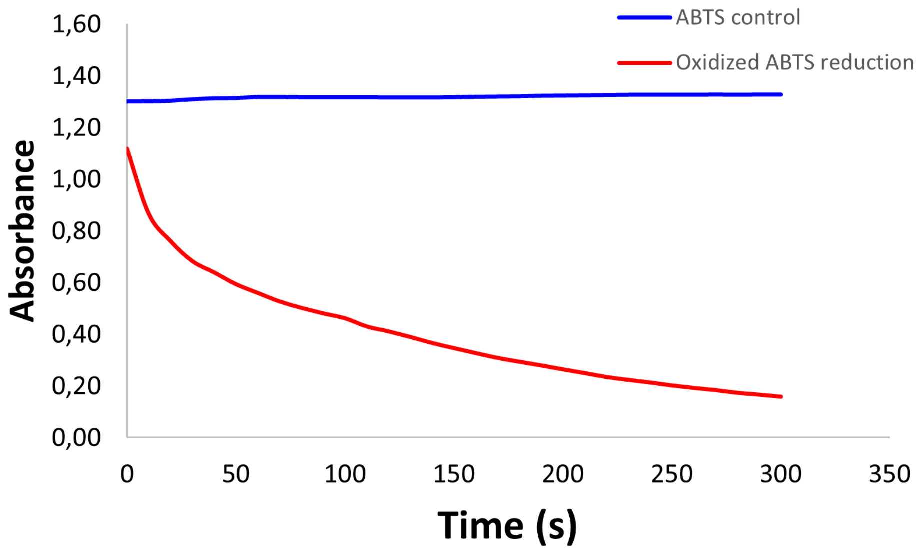

Figure S7. UV-Vis absorbance at 410%nm showing reduction of oxidized ABTS by uric acid versus control.

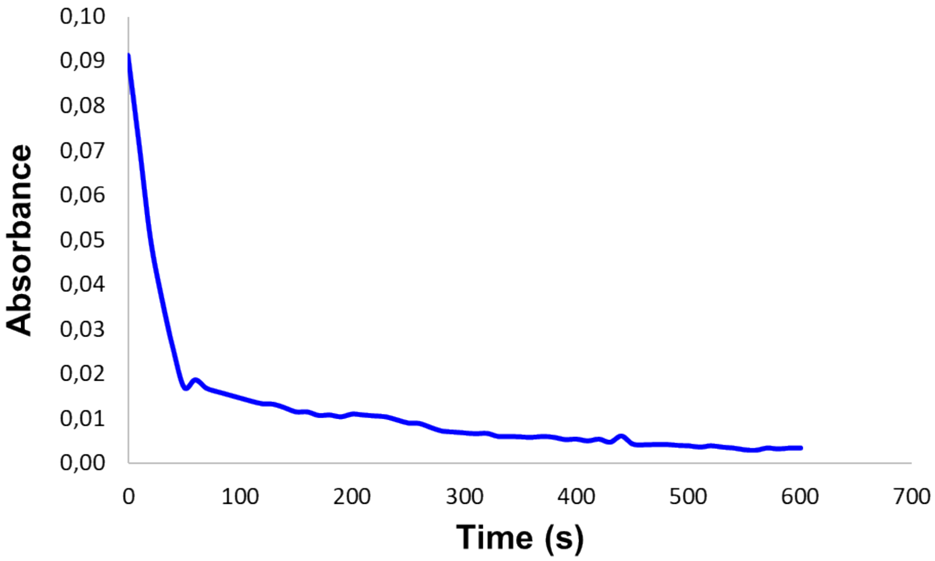

Figure S8. Activity of free enzyme cascade measured by ABTS test at 410%nm over time for a reaction mixture containing 100%µL of HRP (20%U/mL), 100%µL of XO (0.5%U/mL), 1%mL of 0.2%mM ABTS, and 1%mL of 0.37%mM hypoxanthine at room temperature (23%˚C).

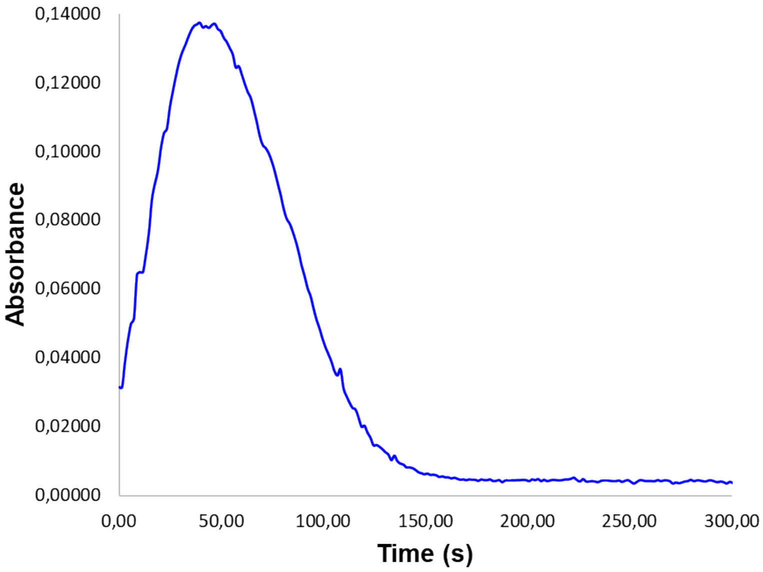

Figure S9. Activity of free enzyme cascade measured by ABTS test at 410%nm over time using reduced HRP volume. Reaction conditions: 1%mL of 0.2%mM ABTS, 1%mL of 0.05%mg/mL hypoxanthine (≈0.37%mM), 100%μL of XO (0.5%U/mL), and 20%μL of HRP (20%U/mL). The experiment was carried out at 23%˚C.

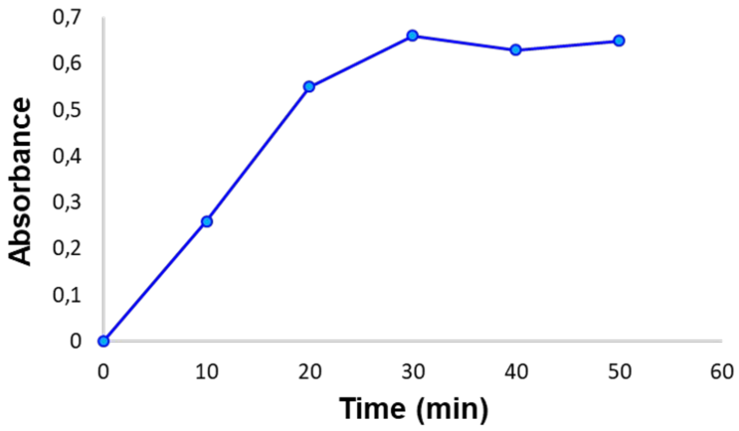

Figure S10. Activity of immobilized enzyme cascade measured by ABTS test at 410 nm over time. Reaction conditions: 1%mL of 0.2%mM ABTS, 1%mL of 0.05%mg/mL hypoxanthine (≈0.37%mM), 50 mg of XO, and 50 mg of HRP. The experiment was carried out at 23%°C.

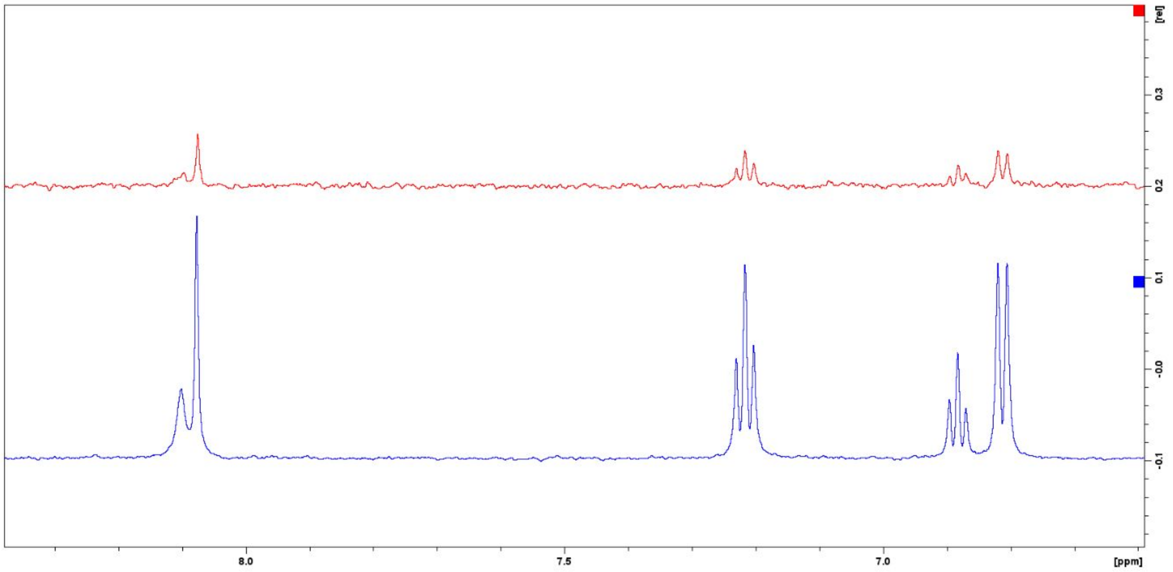

Figure S11. Degradation of phenol by core shell immobilized perlite-HRP-XO cascade: A. initial phenol (blue) and phenol after 24 hours of interaction with perlite-HRP-XO (red). The NMR sample solution was H2O:D2O 90%:10%.

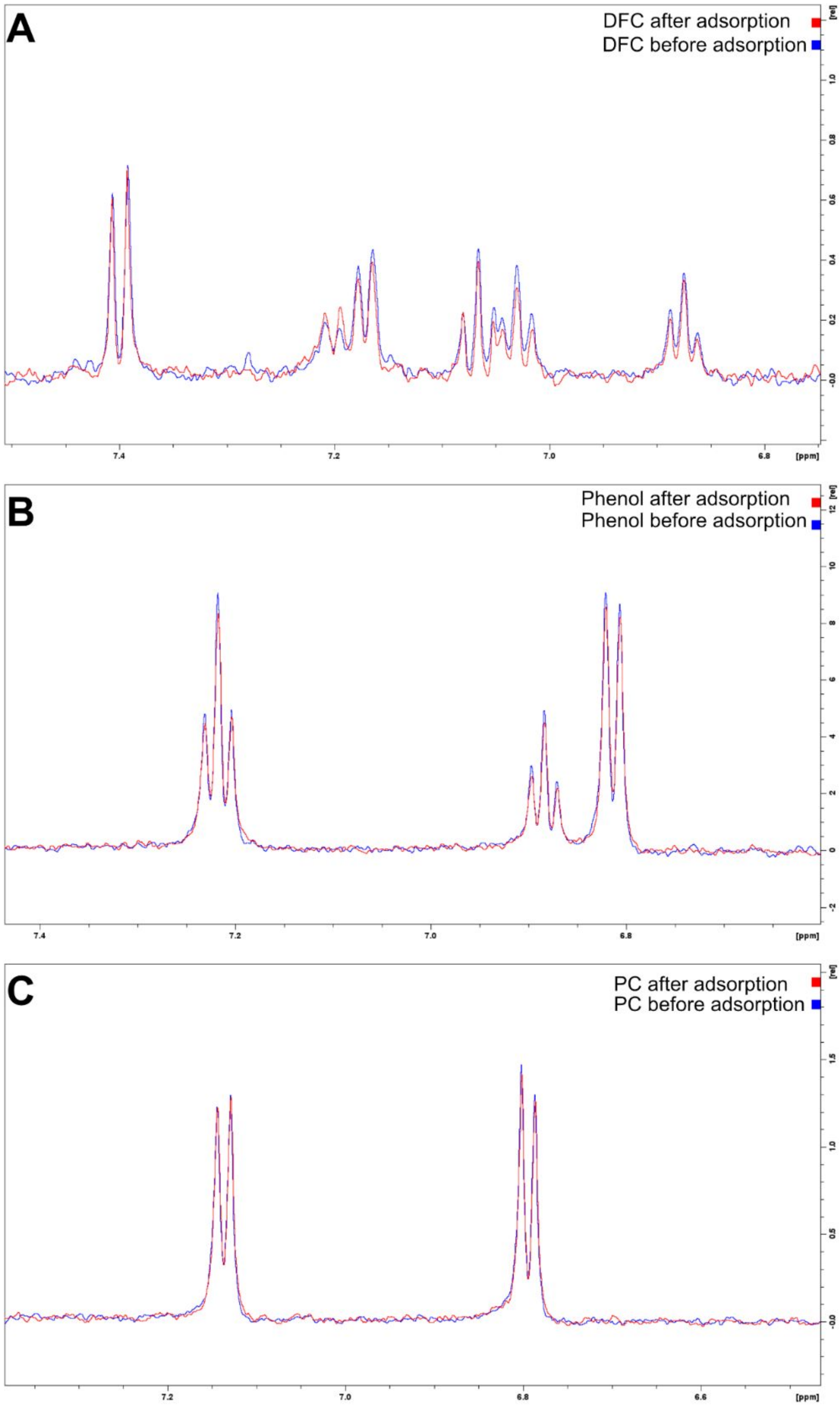

Figure S12. Control experiments with silica-coated perlite (without enzymes): A. initial DFC (blue) and DFC after 24 hours of interaction with silica-coated perlite (red); B. initial phenol (blue) and phenol after 24 hours of interaction with silica-coated perlite (red); C. initial PC (blue) and PC after 24 hours of interaction with silica-coated perlite (red). The NMR sample solution was H_2O:D_2O 90%:10%.
